# On the Revival of the Taliacotian Art

**Published:** 1816-04

**Authors:** 


*For the London Medical and Physical Journal.*

*On the Revival of the Taliacotian Art;* by J.V. *of Cornwall.*

THE object of the following letter is to offer a few remarks on an operation which has lately been performed in England under the title of the Nasal Operation, with the design of restoring the deformity of a lost Nose. Mr. Carpue, Professor of Anatomy and Surgery in London, has performed it twice, and in both instances with success. His method differs from that practised by the celebrated Taliacotius,* in the flesh being taken from the forehead instead of the arm. This operation has been performed in India for many years by one of the casts of the natives. If men like these are capable of an operation requiring great accuracy, how much more are the practical surgeons of England adequate to it, who are, in general, men of science, erudition, and professional skill.

The reason for which this operation has been neglected may be partly owing to most of the cases which occur in England being the consequence of disease: now, in India it is diametrically opposite, for it is well known that many crimes in that country are punished by cutting off the nose,

---

* This antient surgeon appears to have the merit of introducing some of the first improvements in that part of surgery which relates to adhesion; but, through the extravagance of some of his followers, and the irresistible operation of the wit and humour of his satirists, his name has become irrevocably connected with ideas of ridicule.—Med. and Phys. Journ. vol. ix.

He wrote two works, entitled—

Taliacotii (Casp.) de Curtorum Chirurgia, Fol. Venet, 1597.

———————— Chirurgia nova Curtorum sive de Narium, Aurium, Labiorumque Defecta, &c, 8vo. Francof. 1598.

ears, ears, &c. This circumstance may make a material difference in the success attending the operation, for a diseased part is not so likely to heal by the first intention as a healthy one.

Butler, in his Hudibras, places this operation in a ludicrous light.

> Sic adscititios nasos de clune torosi
> Sectoris, doctâ secuit Talicotius arte:
> Qui potuêre parem durando æquare parentem.
> At postquam fato Clunis computruit, ipsum
> Una sympathicum cœpit tabescere Rostrum.*

In Heister's Surgery, a chapter is written on the subject of artificial noses, in which he says, that what is proposed by Taliacotius, in his work entitled Chirurgia Curtorum per Insitionem, is, for want of later experiments and observations, judged to be impracticable, and without foundation, by our modern surgeons. He then observes, when this member is lost, we must supply the defect by an artificial nose of silver or wood, unless, by being on the spot, you can instantly replace and conjoin the real nose just separated, either by suture or emplasters. Such an artificial nose, painted to the life, and adapted by proper springs and screws, may render the accident and deformity imperceptible.

In offering these slight hints, I am not intending to inculcate that the nasal operation is of as much consequence as that for the relief of a strangulated hernia, or those for the depression or extraction of the cataract—by no means; but that, like them, it requires some share of manual dexterity, so that the surgeon who should attempt this operation ought to possess all the qualifications required by Celsus, "manu strenua stabili, nec unquam intremiscente, animo intrepidus immisericors."

In writing the above, I fear I have trespassed too much on your valuable pages, and shall now conclude with the utinam—

> Quod floreat ars chirurgica
> Floreantque ejusdem peritissimi professores.

---

* The Editors will thank their learned correspondent to inform them where the above Latin translation is to be met with. The following are the lines in Butler's Hudibras:—

> Thus learned Taliacotius from
> The brawny part of porter's bum,
> Cut supplemental noses, which
> Lasted as long as parent breech;
> But, when the date of Nock was out,
> Off dropt the sympathetic snout.

We shall shortly notice Mr. Carpue's account of his success in this operation.

